# Screened selection design for randomised phase II oncology trials: an example in chronic lymphocytic leukaemia

**DOI:** 10.1186/1471-2288-13-87

**Published:** 2013-07-03

**Authors:** Christina Yap, Andrew Pettitt, Lucinda Billingham

**Affiliations:** 1 MRC Midland Hub for Trials Methodology Research, College of Medical and Dental Sciences, University of Birmingham, Birmingham B15 2TT, UK; 2 Molecular and Clinical Cancer Medicine, University of Liverpool, Liverpool L69 3GA, UK; 3 Cancer Research UK Clinical Trials Unit, University of Birmingham, Birmingham B15 2TT, UK

**Keywords:** Randomised Phase II, Selection Design, Screening Design, Play-the-Winner, Oncology, Moderate Sample Sizes

## Abstract

**Background:**

As there are limited patients for chronic lymphocytic leukaemia trials, it is important that statistical methodologies in Phase II efficiently select regimens for subsequent evaluation in larger-scale Phase III trials.

**Methods:**

We propose the screened selection design (SSD), which is a practical multi-stage, randomised Phase II design for two experimental arms. Activity is first evaluated by applying Simon’s two-stage design (1989) on each arm. If both are active, the play-the-winner selection strategy proposed by Simon, Wittes and Ellenberg (SWE) (1985) is applied to select the superior arm. A variant of the design, Modified SSD, also allows the arm with the higher response rates to be recommended only if its activity rate is greater by a clinically-relevant value. The operating characteristics are explored via a simulation study and compared to a Bayesian Selection approach.

**Results:**

Simulations showed that with the proposed SSD, it is possible to retain the sample size as required in SWE and obtain similar probabilities of selecting the correct superior arm of at least 90%; with the additional attractive benefit of reducing the probability of selecting ineffective arms. This approach is comparable to a Bayesian Selection Strategy. The Modified SSD performs substantially better than the other designs in selecting neither arm if the underlying rates for both arms are desirable but equivalent, allowing for other factors to be considered in the decision making process. Though its probability of correctly selecting a superior arm might be reduced, it still performs reasonably well. It also reduces the probability of selecting an inferior arm.

**Conclusions:**

SSD provides an easy to implement randomised Phase II design that selects the most promising treatment that has shown sufficient evidence of activity, with available R codes to evaluate its operating characteristics.

## Background

In recent years, there has been an increase in the number of new potential therapies for treatment for chronic lymphocytic leukaemia. In this setting where the number of patients is limited and there are several potentially promising treatments, the selection of efficient and reliable statistical methodologies to evaluate these new treatments in Phase II is vital.

### Motivating example

The design proposed in this paper is motivated by a trial in Chronic Lymphocytic Leukaemia (CLL). CLL is a cancer of the white blood cells in which there is an excess number of poorly functioning lymphocytes in the circulating blood. It is the most common adult leukaemia in the Western World. The study population includes patients with high risk CLL, incorporating those cancers with the genetic defect of p53 deletion, as well as those who have failed standard chemotherapy. Conventional therapy is not curative and thus treatment options are limited. There is no agreed standard care and there is significant morbidity and mortality in this group of patients. A recent Phase II study, CLL206, evaluated the CAM-PRED (alemtuzumab+high-dose methylprednisolone) regimen and produced an impressive complete response with complete bone marrow recovery rate, CR, of 23% [1]. However, effects were short-lived with median progression free survival of only 7 months. There was also a high rate of grade 3–4 infections and steroid-related toxicity.

Building on from the CAM-PRED trial [1], it was of interest to investigate two potential new treatment regimens, CAM-DEX (Alemtuzumab+Dexmethasone) and CAM-DEX-REV (Alemtuzumab+Dexmethasone+Lenalidomide). The primary efficacy (also known as activity in Phase II setting) outcome of interest is complete response rate with complete marrow recovery (where all signs of leukaemia have disappeared) as defined by the IWCLL response criteria [2]. Replacement of high dose methylprednisolone (PRED) with oral steroids, dexamethasone (DEX) in the first treatment, CAM-DEX, is believed to reduce infection and steroid related toxicity whilst maintaining efficacy. Lenolidomide is included in the second treatment, CAM-DEX-REV, as it has established activity in CLL and has a favourable toxicity profile. The CAM medication is administered via the subcutaneous route throughout the study as opposed to the CAM-PRED trial where it was given intravenously for the first month and then subcutaneously thereafter. The change is proposed as subcutaneous administration is easier and produces fewer side effects, whilst demonstrating comparable results. CAM-DEX is expected to be comparable to CAM-PRED while CAM-DEX-REV is believed to produce a higher complete response rate.

The absence of any effective standard therapy and the limited number of available patients in this study population led us to consider comparison to historical controls’ complete response rates, which in this case, is close to null for patients that are left untreated or undergo palliative care. In this trial, there are two new treatment regimens to be evaluated. To improve patient comparability, we chose a randomised trial design rather than conduct two separate trials. There are various approaches that have been used for Randomised Phase II Designs, namely Parallel Simon’s 2 stage Design [3], Simon-Wittes-Ellenberg Selection Strategy (SWE) [4], Jung and George’s Randomised Phase II [5], typical methods in Phase III design with relaxed Type 1 and Type 2 errors [6] and a Bayesian Selection Strategy [7,8].

In the approach with parallel Simon’s 2 stage, the required number of patients for the first stage is first randomised into each arm. If there is insufficient efficacy, recruitment to the arm will stop, whilst if there is sufficient efficacy, a further group of patients will be recruited for the second stage. At the end of the trial, if there is sufficient efficacy, the treatment arm will be considered active. The advantage of using this design is that it allows for early termination if it does not show promising efficacy at the interim stage. If both arms are shown to be active, an ad hoc decision can be taken to select the arm with the highest complete response rate. However, this would unfortunately be an unplanned decision as we have not incorporated the selection between arms in the design.

SWE [4] has grown in its popularity as an established randomised Phase II methodology. The approach is based on the statistical method of ranking and selection and is not based on the conventional hypothesis testing. It considers randomizing *n* patients to each of the treatment arms through a single stage and picking the winner, the arm with the largest estimated response rate among them. This design is attractive as it is very simple and only requires moderate sample sizes to provide a high probability of correctly selecting a superior treatment for further testing in Phase III when such a treatment exists. However there are several disadvantages with this design. Firstly, it controls the false negative rate, but makes no attempt to control the false positive rate, which in fact is always 100% [9]. In the case where both arms have equal response rates, you would always be (incorrectly) selecting one treatment as superior. It is only appropriate in settings whereby it is not crucial that the superior treatment arm is selected if the activity rates are fairly similar. Secondly, it will also select an arm with the highest response rate even if none of the arms give response rates that are of clinical significance.

This led us to propose the Screened Selection Design (SSD), which combines the two conventional Phase II methodologies, Simon’s 2 stage and SWE, and aims to improve and minimise the shortfalls of the two approaches. In addition, a variant of this design, the Modified Screened Selection Design also allows the user the flexibility to only select the active treatment arm with the highest response rate if the activity rate is greater than the other arm by a specified clinically-relevant value (hence, reducing the chance that you would select an inferior arm). This would allow other criteria, e.g. safety and toxicity, quality of life or secondary efficacy outcome measures to be considered in the final selection process, which is generally what happens in clinical practice when we see two very similar success rates in treatments. A similar idea was proposed by Sargent and Goldberg [10], which extends the SWE to consider other factors when there is only a small difference between the activity rates.

The remainder of the paper is organised as follows: The Methods section describes the proposed design and an alternative approach via a Bayesian selection strategy[7]. This is followed by the Results section, which presents a simulation study to evaluate the operating characteristics of the proposed design, and compares it with the conventional SWE and a Bayesian selection strategy approach. It also presents a strategy for practical implementation of the design and evaluates the performance of the SSD for a wide range of activity rates. This is followed by Discussion and Conclusion.

## Methods

### Screened selection design (SSD)

The Screened Selection Design involves first applying Simon’s two-stage design [3] in each of the two parallel experimental arms, which allows for initial determination of efficacy and early stopping for futility in any of the arms. If there are an insufficient number of responses in the first stage, recruitment will not continue for that particular arm. Otherwise, the study proceeds to stage 2 to randomize further patients to each arm. The second segment of the study involves the play-the-winner selection strategy as proposed by Simon, Wittes and Ellenberg [4], which only applies if results from both arms are found to be positive. SSD will recommend the treatment arm with the highest response rate. An extension of the SSD, the modified SSD only selects the treatment arm with the highest response rate if the activity rate is greater by a specified clinically-relevant value.

In the CLL example, a desirable complete response rate is 20%. If we were to apply the SWE design to this trial, we would require 29 patients per treatment arm to allow at least 90% chance of correctly selecting the better arm when the superior arm is 15% higher (i.e. 20% vs. 35%). In our proposed SSD, we opt to keep the sample size the same as that in SWE at 29 per arm, and explore its operating characteristics in comparison to SWE using simulations (see Section 3).

**Figure 1:**
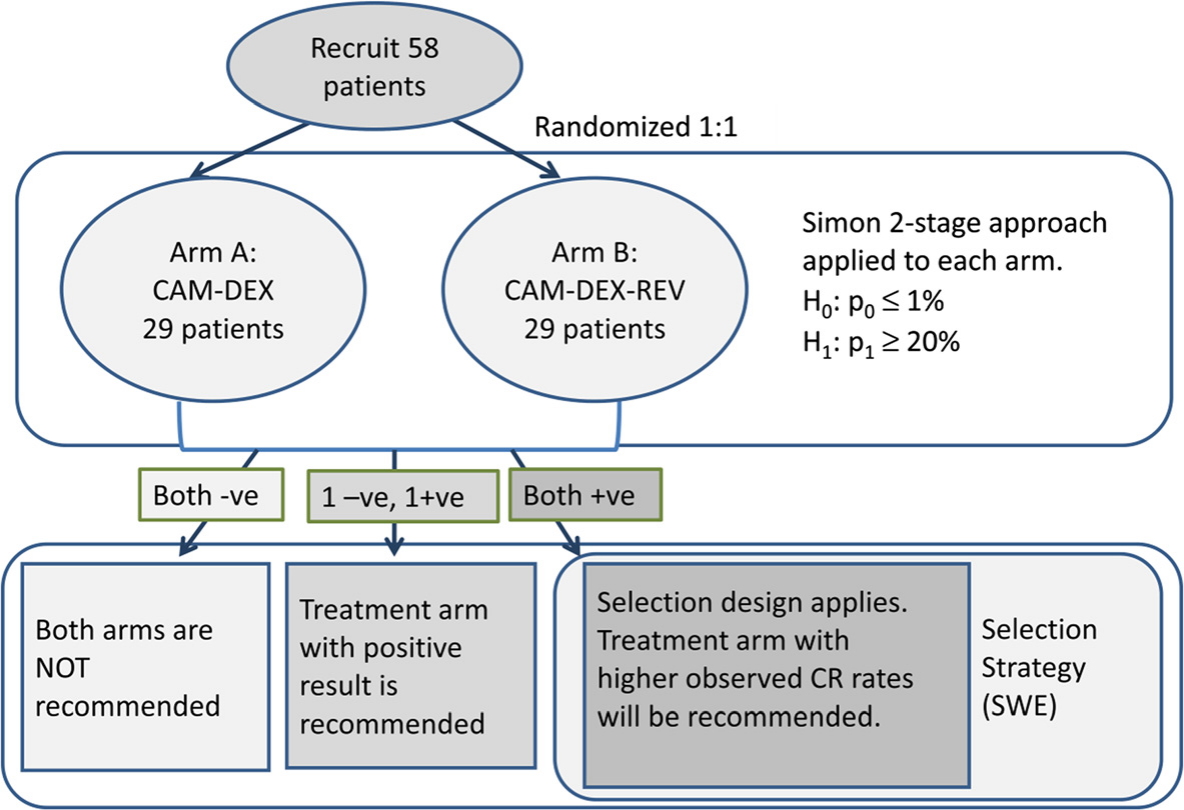
**Screened selection design.** A flow diagram of the Screened Selection Design, as applied to the CLL trial with two randomised treatment arms, CAM-DEX and CAM-DEX-REV, with 58 patients.

With N set at 29 and desiring a CR rate of at least 20% for an active arm in comparison to an undesirable CR rate of 1% or less (with only palliative care) and nominal alpha and beta set at 0.05, 14 patients would be randomized to each arm for the first stage in the Simon’s two-stage design. If there is no CR, recruitment to the particular arm will stop. Otherwise, if there is at least 1 CR, 15 additional patients will be accrued for a total of 29 patients. The null hypothesis that the CR rate is undesirable at ≤ 1% will be rejected and the treatment arm would be considered active if there are at least 2 out of 29 patients who had CR. This design yields an exact type I error rate of 0.026, and a power of 0.951 for the first segment when the true CR rate is 20%. (Note: This is one possible 2 stage design for N = 29 [11], which is also the optimal design under both nominal alpha and beta constraints of 0.05.) If both arms are active, SSD then selects the arm with the highest response rate. This is illustrated in Figure 1.

The modified SSD incorporates an extra feature where in the case when the observed rates of both effective arms are fairly similar, e.g. less than a specified difference, say 5%, we would not select either arm only based on the primary outcome measure. Other criteria can be considered to aid the selection.

SSD inherited the attractive features of being able to check for efficacy at the interim stage and only allowing arms that have been shown to be effective to undergo the selection stage to be considered for further testing. In this way, there should be a higher probability that the final selected arm is not only superior but also active.

### Comparison to a Bayesian approach

We compare the performance of the SSD and Modified SSD with the Bayesian Selection Strategy proposed by Estey and Thall [7] via a simulation study in Section 3. The working of the Bayesian Selection Strategy is similar to that of the SSD in that the initial segment includes checking for activity, allowing for early stopping for futility, before selecting the superior arm. However, this is approached from the Bayesian framework. The method will compare the posterior probabilities of response for patients in the trial on treatment A and B with data using CAM-PRED in the CLL206 trial [1], denoted treatment S. Using the same sample size of 29 per arm, we apply this design to the CLL trial and evaluate its operating characteristics.

The prior probability for the treatment S is taken as beta (9,30) with 23% CR (i.e. 9 CRs and 30 non-CRs) [1]. We assume a prior for each experimental arm A and B as beta (0.4615, 1.5385), which is a weakly informative prior, that has the same mean (i.e. mean CR of 23%) as treatment S in CAM-PRED but with a low equivalent sample size of 2, as recommended in [12].

The Bayesian Selection Strategy (BSS) will stop the trial if we are 90% sure that the experimental arm, E is less responsive than treatment S by 3% (desirable CR for E is at least 20%), i.e.

$${P(\theta }_{E}<\;\theta_{S}\;–\;0.03\;|\;\mathrm{data})>0.9$$

where θ_E_ and θ_S_ are the probabilities of response in experimental arm (A or B) and treatment S respectively and using the same notation as [12] with δ = − 0.03 and π* = 0.9.

Using the above values for δ and π*, with implementation of stopping rules after every cohort of 10 patients, this gives rise to stopping boundaries of ≤ 0/10 and ≤ 1/20. An arm is selected if it accrues to the maximum sample size of 29 and has the highest observed CR rate. The stopping boundaries are obtained using the Multc Lean software [12] and could be altered to make them more or less protective by changing the values of δ and π*.

A subtle difference to the working of SSD and BSS is that in SSD we would require an arm to be deemed effective (at least 2 CRs) before it is being allowed to be in the competition to be selected amongst all the other effective arms, whereas the BSS only requires the maximum sample size to be reached before the selection process.

## Results

### Comparison of results to conventional SWE

We explored the operating characteristics of our proposed designs, SSD and modified SSD, with 1 million replications, and compared it with SWE and the Bayesian Selection Strategy using a fixed sample size of 29 per arm for each of these designs. The operating characteristics describe the three designs’ average behaviour under various reasonable clinical scenarios in terms of overall selection probabilities of Arm A, Arm B or No Selection. The scenarios included settings whereby both arms have either the same or different response rates within a relevant range that is applicable for the CLL trial.

No arm is selected for SSD if both arms are negative after the 1st segment. This also applies to the Modified SSD, but in addition, it also includes no selection of arms when the observed difference is less than *d* units (which is taken as 5% in this example). Probability of selecting no arm for SWE is always 0, as it would always select an arm even if both arms are ineffective or equivalent. The results of the simulation study are displayed in Table 1.

**Table 1:** Simulation study to evaluate and compare performance of SSD, Modified SSD, SWE and Bayesian selection strategy

**Scenario**	**True CR (pA,pB)**	**Simulated overall selection probabilities**											
		**SSD**			**Modified SSD**			**SWE**			**Bayesian selection strategy**		
		**Arm A**	**Arm B**	**No Arm**	**Arm A**	**Arm B**	**No Arm**^**+**^	**Arm A**	**Arm B**	**No Arm**	**Arm A**	**Arm B**	**No Arm**
**1**	**(0.01,0.01)**	0.025	0.025	**0.949**	0.025	0.025	**0.950 (0.001)**	0.500	0.500	**0**	0.013	0.013	**0.974**
**2**	**(0.1,0.1)**	0.455	0.454	**0.091**	0.311	0.311	**0.379 (0.287)**	0.500	0.500	**0**	0.383	0.383	**0.234**
**3**	**(0.2,0.2)**	0.500	0.498	**0.002**	0.320	0.320	**0.359 (0.357)**	0.500	0.500	**0**	0.490	0.490	**0.019**
**4**	**(0.3,0.3)**	0.500	0.500	**0.000**	0.334	0.335	**0.331 (0.331)**	0.500	0.500	**0**	0.500	0.500	**0.001**
**5**	**(0.01,0.03)**	0.023	0.167	**0.810**	0.021	0.164	**0.815 (0.004)**	0.315	0.685	**0**	0.012	0.094	**0.894**
**6**	**(0.01,0.2)**	0.002	**0.950**	0.048	0.001	**0.947**	0.051 (0.003)	0.003	**0.997**	0	0.002	**0.864**	0.134
**7**	**(0.20,0.35)**	0.100	**0.900**	0.000	0.042	**0.805**	0.154 (0.154)	0.099	**0.901**	0	0.104	**0.894**	0.002
**8**	**(0.2,0.4)**	0.047	**0.953**	0.000	0.017	**0.897**	0.086 (0.086)	0.046	**0.954**	0	0.049	**0.950**	0.001

The table displays a simulation study to evaluate the operating characteristics of Screened Selection Design (SSD) and Modified Screened Selection Design (Modified SSD), and compare it to Simon-Wittes-Ellenberg Selection Strategy (SWE) and Bayesian Selection Strategy, based on 1 million replications. The overall probabilities of selecting Arm A, selecting Arm B and selecting neither arm (No Arm) for each design under various scenarios are presented. Scenarios 1–4 denote situations when the rates at both arms are the same, whereas Scenarios 5–8 denote situations when the rates are different. The values in ***bold*** indicate the correct selection probabilities under specific scenario. CR: Complete Response rate, pA: true CR rate for Arm A, pB: true CR rate for Arm B. ^+^The values in parenthesis for Modified SSD indicate the probability that neither arm is selected based on the primary endpoint when the observed difference is less than the clinically-relevant value of 5%. This then directs the decision making process to other additional clinical factors to choose between the two active arms.

As expected, when the true CR rates are below clinical significance (Scenarios 1 and 5), the performance of SSD and Modified SSD are far superior to SWE as they correctly select none of the arms most of the time compared to 0% for SWE. Modified SSD does substantially better by selecting neither arm based on the primary endpoint when both CR rates are desirable but are the same (Scenarios 3 and 4), with over 33% increase in identifying arms that have less than 5% difference in response rates. Pre-specified secondary criteria could be used to select between the two active arms. It has the lowest probability of selecting the inferior arm when the CR rates of the two arms are different (in Scenarios 5–8).

In terms of correctly selecting an effective arm which is superior (in Scenario 6–8), SSD is lower than SWE in Scenario 6 where it loses 4.8% in the first segment to selection of neither arms for inactivity. However it is comparable to SWE when both arms are active. Probability of correct selection for Modified SSD is lower though it still performs reasonably well.

### Comparison of results to a Bayesian selection design

From the simulation results in Table 1, the Bayesian Selection design is comparable to that of SSD. It has a higher probability of correctly selecting no arm when both are inactive, but has a lower probability of correctly selecting a superior arm compared to SSD. Its working characteristics of Bayesian Selection Strategy can however be altered by changing the values in (1) to make it more or less protective against different error criteria. As in SSD, it could be easily extended to incorporate selection of superior arm only if it is greater than the other arm by *d* %.

From Table 2, we can observe that the sample size required for the Bayesian Selection Strategy is generally lower when the arms are ineffective compared to SSD. However, in the case when the arms are effective, SSD performs better in recruiting a higher mean number of subjects. As expected, both approaches provide a substantial reduction in mean sample size particularly when the arms are inactive.

**Table 2:** Mean number of subjects required in SSD/Modified SSD compared to Bayesian selection strategy in the CLL trial example

**Scenario**	**True CR (pA,pB)**	**Average number of subjects**			
		**SSD/Modified SSD**		**Bayesian selection strategy**	
		**Arm A**	**Arm B**	**Arm A**	**Arm B**
1	(0.01,0.01)	16.0	16.0	11.1	11.1
2	(0.1,0.1)	25.6	25.6	21.2	21.2
3	(0.2,0.2)	28.3	28.3	26.7	26.7
4	(0.3,0.3)	28.9	28.9	28.4	28.4
5	(0.01,0.03)	16.0	19.2	11.1	13.5
6	(0.01,0.2)	16.0	28.3	11.1	26.7
7	(0.20,0.35)	28.3	29.0	26.7	28.8
8	(0.2,0.4)	28.3	29.0	26.7	28.9

The table displays the mean number of subjects required for the Screened Selection Design (SSD)/Modified Screened Selection Design (Modified SSD) and the Bayesian Selection Strategy, obtained from the same simulation study as presented in Table 1. Number of subjects required in SWE is always 29 per arm, whereas the other two approaches, allowing for early stopping due to futility, have a lower mean number of subjects when the arms are not active.

### Practical implementation of SSD

In practice, when a trial is designed using SSD, we would assess the operating characteristics (OCs) under various design parameters and reasonable clinical scenarios via simulations (as in Table 1), and utilise these as a basis for choosing design parameters that have desirable OCs. The R code to evaluate the OCs for SSD is freely available (see Additional files 1 and 2).

The focus so far has been based on a worked example of a trial with 2 experimental arms which are believed to have rates that are clinically desirable, but one having a much more superior CR rate. In this example, the undesirable rate, p0, is 1%, with CR in Arms A and B at pA = 20% and pB = 35%. Next, let us consider the performance of the SSD for different response rates with various design parameters where the undesirable rates p0 range from 1% to 65% and the response rates in the experimental arms are at least 15% higher. Using the same sample size as that required in SWE with alpha constrained to a specified value, we evaluate the overall probability of correctly selecting the superior arm.

A strategy we adopted is detailed below:

(1) First, we determine the sample size, n, that is required in SWE for pA and pB. This can be easily obtained from Table 3 in [4], clinfun package in R [13] or specialist software packages such as PASS.

(2) Using the same sample size in (1), we aim to find suitable alpha and beta to screen for activity in each arm for the first segment of SSD with Simon’s two-stage design.

(a) We choose to constrain alpha to at most 20% (i.e. we are happy for a 20% or less chance of incorrectly identifying a treatment as effective when it is not).

(a) To find suitable r1 and r, we first start with nominal beta = 0.05 and nominal alpha = 0.2, and select the optimal 2-stage design with sample size n. However, if no such design exists, go to (c).

(a) Keeping alpha at 20%, one can increase beta by steps of 1% till you obtain a design with sample size n.

(3) Evaluate the overall probability of correctly selecting the superior arm.

**Table 3:** Sample size and overall probability of correctly selecting a superior arm in SSD for different response rates based on 1 million replications

**Scenario**	**Undesirable rate, p0**	**Desirable rates**		**n**	**Nominal alpha (exact alpha)**	**Nominal beta (exact beta)**	**First segment (Simon’s 2 stage)**		**Overall probability of correctly selecting a superior arm**
		pA^*^	**pB**				**r1/n1**	**r/n**	
1	1%	20%	35%	29	0.05 (0.026)	0.05 (0.049)	0/14	1/29	0.900
2	5%	20%	35%	29	0.2 (0.169)	0.06 (0.059)	0/18	2/29	0.901
3	10%	30%	45%	35	0.2 (0.187)	0.05 (0.049)	2/19	4/35	0.903
4	15%	30%	45%	35	0.2 (0.197)	0.15 (0.122)	5/28	6/35	0.903
5	20%	40%	55%	37	0.18 (0.177)	0.05 (0.048)	3/19	9/37	0.902
6	25%	40%	55%	37	0.2 (0.191)	0.14 (0.138)	4/22	11/37	0.902
7	30%	50%	65%	36	0.2 (0.160)	0.07 (0.070)	5/21	13/36	0.901
8	35%	50%	65%	36	0.2 (0.198)	0.2 (0.191)	9/24	14/36	0.900
9	40%	60%	75%	32	0.2 (0.159)	0.1 (0.100)	8/21	15/32	0.900
10	45%	60%	75%	32	0.2 (0.198)	0.21 (0.204)	12/25	16/32	0.900
11	50%	70%	85%	26	0.2 (0.161)	0.13 (0.128)	7/16	15/26	0.904
12	55%	70%	85%	26	0.2 (0.194)	0.23 (0.230)	10/20	16/26	0.903
13	60%	80%	95%	16	0.2 (0.163)	0.21 (0.209)	4/8	11/16	0.904
14	65%	80%	95%	16	0.2 (0.191)	0.36 (0.357)	7/10	11/16	0.901

p0 : response proportion of a poor drug.

pA : response proportion of Arm A.

pB : response proportion of Arm B.

alpha : probability of rejecting p ≤ p0 when this is true (exact alpha provided in parenthesis).

beta : probability of rejecting p ≥ pA^*^ when this is true (exact beta provided in parenthesis).

pA is taken as the minimum response rate of a good drug.

n1 : no. of subjects required for Stage 1 in Simon’s 2 stage.

r1 : maximum number of successes in which will terminate trial.

r : maximum number of successes at the end of Stage 2 not to warrant further investigation.

Simon 2 stage optimum design with r1, n1, and r, which satisfies the (nominal) error constraints, is selected for the fixed sample size of n for each scenario. The alpha and beta rates are specific to each arm in the first segment, and not for the trial as a whole. Following the strategy as described above, if we have two experimental arms at 30% and 45%, a sample size of 35 is required for SWE to obtain an overall probability of correct selection of superior arm of at least 90%. Using n = 35 with undesirable rate assumed at 10%, and setting nominal alpha and beta at 0.2 and 0.05 respectively, the optimal 2-stage design is r1/n1 = 2/19 and r/n = 4/35. This gives an overall probability of correctly selecting the superior arm as 0.903 for SSD. This is given as Scenario (3) in Table 3, along with other scenarios of different rates of p0, pA and pB.

The results from Table 3 demonstrate one of the most appealing properties of SSD. We are able to retain the sample size as required in SWE, and still be able to obtain at least 90% probability of correctly selecting the superior arm, with the added benefit of screening out inactive arms (alpha ≤ 0.2).

Table 3 provides a reference to possible Screened Selection Designs that can be used for different response rates. They are examples rather than defaults. As noted earlier, it is possible to choose different alpha and beta (and hence different r1 and r) for the same sample size, as is relevant to a particular trial.

## Discussion

We proposed an easy to implement randomised Phase II design which performs a dual task; to first evaluate activity before selecting the most promising treatment regimen for further testing in Phase III, for moderate sample sizes.

As this design builds on the existing selection strategy by [4], it is particularly useful in settings where only limited number of subjects is available. If however, the pool of patients is much larger, we could consider a typical Phase III type design but with relaxed alpha and beta errors [6] with alpha (two-sided) and beta of 0.2. This would require 182 patients (91 per arm) to determine if there is a difference between the independent proportions of 0.2 and 0.35 based on Fisher’s Exact test. If a test is carried out with one-sided alpha, this would require 126 patients (63 per arm), which is still a much larger sample size compared to designs using selection strategy, as in SWE.

Conventional statistical designs require much larger number of patients, as they select a treatment as superior only when the observed data are incompatible with the hypothesis that the treatments are equal. In the approach suggested by Jung & George [5], they proposed carrying out Simon’s 2 stage in the initial part, before selecting the superior arm based on hypothesis testing. For a fixed probability of correctly selecting the superior treatment, this would again require a larger sample size than our proposed design of SSD based on selection theory. Alternatively, for a fixed sample size, the probability of correctly selecting the superior treatment is substantially higher with Modified SSD compared to Jung and George’s design. This can be illustrated by considering Example 4 from their paper [5] which for a fixed sample size of 45 patients designed to compare hypothesized response rates of 15% vs 35% the probability of correctly selecting the superior treatment when the true rates are 25% vs 45% is 0.927 with modified SSD compared to 0.639 with Jung and George.

On the other hand, selection theory designs always selects a treatment as superior, even if they are actually equivalent. This design flaw is carried over into the SSD design so that if both treatments have rates that are above undesirable activity but are identical (Scenarios 2–4 in Table 1) then the design will still select an arm that is superior. With the addition of a decision rule of observing a superior difference of at least 5%, the Modified SSD does better by at least 28% in such settings, in selecting neither arms based on the primary outcome. Instead, it directs the decision to additional factors to determine the selection of the superior arm. There is however still a reasonably high chance that the SSD and Modified SSD will select an arm as superior even if both arms are equivalent or similar, but it is important to note that the objective of such randomised selection trials is not to make a definite conclusion about the superiority of a treatment compared to another. The primary aim of such designs is to ensure that there is a low probability that the inferior treatment will be carried forward to a phase III trial.

The factors that determine the choice between SSD and Modified SSD depend on the clinical setting. Both are designed to remove ineffective arms before selecting the superior one. In the setting where it is not crucial for the chosen arm to be truly superior, and in fact could be slightly inferior, SSD can be used. However, in the case where it is important that there is a high probability that the selected arm is superior, it would be more appropriate to use Modified SSD. By allowing no arm to be selected when the rates are fairly similar based on the primary outcome measure, this design enables other pre-planned clinical criteria (as stated in protocol) that are relevant to the decision process, to make the distinction between the two active arms. This is similar to settings when we would consider choosing either SWE or Sargent-Goldberg screening design [10].

Using the same sample size as in SWE, the proposed approach has the advantage of substantial reduction in the probability of incorrectly selecting an ineffective arm whose rates are not clinically significant (both SSD and Modified SSD) or when no true difference exists between the arms (Modified SSD). As we have observed from the simulation results in the CLL example, the probability of correctly selecting a superior arm is very similar in the SSD and SWE when both arms are effective (i.e. CR ≥ 20%). Whilst SWE only selects the superior arm, SSD only selects the superior arm if it has been shown to be effective. Modified SSD goes a step further from SSD by selecting an effective, superior arm only if it is shown to be superior by a difference of at least *d* % based on the primary endpoint.

The SSD is comparable to the Bayesian approach proposed by Estey and Thall, though the latter has a greater flexibility in intra-arms stopping boundaries. However, it is possible to extend the design of SSD to incorporate more flexible stopping boundaries for futility in the first segment, for instance, using a three stage design which allows for two interim evaluation [14] if this is appropriate. Generally, a two stage design is more common to reduce disruption to the enrolment of the trial while an interim analysis is being carried out.

We illustrated our proposed design for use in trials where there are two potential treatment regimens that have response rates that are hypothesized to be at least 15% better the historical control rates, which is typically used in Phase II trial designs. However, in settings whereby the difference in the rate for the inferior treatment arm and the undesirable rate is small, a much larger sample size might be required for Simon’s 2 stage in the first segment of SSD, within a set constraint of alpha and beta levels. In such cases, it might be useful to consider sample size as the maximum of the two sample sizes required for the two segments and adjust it accordingly by examining the operating characteristics of the design.

The SSD or Modified SSD is most appropriate when it is hypothesized, before the trial, that the potential treatment regimens are effective. If one or neither treatment is believed to be effective, it is unlikely that the trial will move to the selection phase. Liu et al. [15] proposed a comparable design of combining parallel single arm single stage Phase II to screen for activity before selection with SWE. Besides the obvious difference that a single stage is used in theirs compared to a two stage design in SSD, the other main difference is that they considered one of their experimental treatment regimens to have a rate as undesirable as that in the historical controls. They allowed for Bonferroni correction of the type 1 error with alpha set at 0.025 for two treatment arms and power of 0.90. In their example, if CR rate for Arm A is 5% and Arm B is 20% with undesirable and desirable rates at 5% and 20% respectively, there is only a 2.5% (alpha =0.025) chance that Arm A will be deemed positive in the first segment before moving to the 2nd segment for selection, whilst there is a 90% chance (beta = 0.1) that Arm B will be deemed positive. Hence, it is very unlikely that both arms will be positive. In most cases, only Arm B moves to the second segment and is selected by default. Hence, the selection strategy is seldom utilised and the example therefore does not fully illustrate the attractive features of such a design whose intention is select a superior arm after screening independent arms for activity.

Despite the use of randomization and the independent evaluation of activity in each treatment, results from any trial using a SSD or Modified SSD should not be considered definitive. The aim of such a trial design is to select the best active experimental treatment arm to take through to a phase III setting where a formal comparison against the best standard of care will enable decisions regarding the treatment of future patients.

Our proposed design can be used or modified to suit a variety of situations. We have considered Simon’s two stage design to evaluate activity; however, we could also consider tolerability in the first segment to determine that the drug is safe before selecting based on activity. The design can be easily extended to more than 2 arms. Future work includes adapting the design for time to event endpoints, such as progression free survival or overall survival [16,17] and for evaluating joint outcomes of tolerability and activity [18].

## Conclusion

In conclusion, the proposed approach provides a simple and easy to implement randomised Phase II design to select an active and most promising treatment regimen for further testing in Phase III with moderate sample sizes. Simulation studies have demonstrated that it is possible to use the same sample size in SSD as required in SWE, whilst maintaining adequate probability of correctly selecting a superior arm, with the important benefit of reducing the probability of incorrect selection of ineffective arms. Hence, we would strongly recommend considering the use of SSD whenever a selection strategy is appropriate for randomised Phase II design, particularly if there is no strong evidence that the experimental arms are definitely active.

## Abbreviations

SWE: Play-the-winner selection strategy by Simon Wittes and Ellenberg (1985); SSD: Screened selection design; Modified SSD: Modified screened selection design; CLL: Chronic lymphocytic leukaemia; CR: Complete response with complete bone marrow recovery rate; CAM-PRED: Alemtuzumab+high-dose Methylprednisolone) regimen; CAM-DEX: Alemtuzumab+Dexmethasone regimen; CAM-DEX-REV: Alemtuzumab+Dexmethasone+Lenalidomide regimen.

## Competing interests

The authors declare that they have no competing interests.

## Authors’ contributions

CY conceived the study design, programmed simulations and drafted the manuscript. AP contributed to the conception and design. LB contributed to the design, interpretation of the results and commented on the manuscript. All authors read and approved the final manuscript.

## Pre-publication history

The pre-publication history for this paper can be accessed here:

http://www.biomedcentral.com/1471-2288/13/87/prepub

## Supplementary Material

Additional file 1 “Help File for R code for Screened Selection Design.doc”. Click here for file

Additional file 2 “ScreenedSelectionDesign.R”. Click here for file
