# Supplementary material for: Screened selection design for randomised phase II oncology trials: an example in chronic lymphocytic leukaemia
Source: BMC Med Res Methodol. 2013 Jul 3;13:87. doi: 10.1186/1471-2288-13-87 (PMC3726070; doi:10.1186/1471-2288-13-87)
Supplement: Additional file 1 — “Help File for R code for Screened Selection Design.doc”. [file 1471-2288-13-87-S1.docx]

**Help File on R code for Screened Selection Design (SSD)**

**__________________________________________________________________________________**

**SSD.2arms Screened Selection Design (SSD) ________________________________________________________________________________________________________________**

**Description**

SSD.2arms is used to evaluate the operating characteristics of SSD using simulations for a randomised Phase II trial with 2 experimental arms.

**Usage**

SSD.2arms(r1, r, n1, n, p0, p1= NULL, p, diff=0.05, nsim, seed=0802, modified=FALSE)

**Arguments**

| r1 | maximum no. of successes in Stage 1 which will terminate trial |
| --- | --- |
| r | maximum no. of successes in Stage 2 NOT to warrant further investigation |
| n1 | no. of subjects in Stage 1 |
| n | total no. of subjects (Stage 1 + Stage 2) |
| p0 | success probability for historical control arm |
| p1 | success probability for a good drug. Default is pA (refer to p). |
| p | success probability for the 2 experimental arms, p = c( pA , pB ) |
| diff | range of 0 to 1. Default is 0.05, i.e. 5%. Difference must be at least greater than diff before selection of superior arm. Otherwise, none of the arms will be selected. |
| nsim | the number of simulations |
| seed | seed of the random number generator that can be any positive integer |
| modified | controls the summary output. If TRUE, a summary output is printed for Modified SSD. If FALSE, a summary output is printed for SSD. Default is FALSE. |

**Value**

An object consisting of the operating characteristics of the design specified is returned. This contained the following components:

| N | Total Sample Size |
| --- | --- |
| diff | Difference criteria for selection of superior arm in Modified SSD |
| SSD.Arm.A | Overall Selection Probability of Arm A in SSD |
| SSD.Arm.B | Overall Selection Probability of Arm B in SSD |
| SSD.No.Arm | Overall Selection Probability of No Arms in SSD |
| Modified.SSD.Arm.A | Overall Selection Probability of Arm A in Modified SSD |
| Modified.SSD.Arm.B | Overall Selection Probability of Arm B in Modified SSD |
| Modified.SSD.No.Arm | Overall Selection Probability of No Arms in Modified SSD |
| Modified.SSD.No.Arm.diff | Probability where no arm is selected due to lack of clinically-relevant observed differences between the two arms |
| Mean.N.Arm.A | Mean number of subjects in Arm A |
| Mean.N.Arm.B | Mean number of subjects in Arm B |

**Example**

## Scenario 6 in Table 3

source("…./ScreenedSelectionDesign.R")

x.6 <- SSD.2arms(r1=4, r=11,n1=22, n=37, p0 = 0.25, p = c(0.4,0.55), diff=0.05, nsim=1000000, seed=0802, modified = FALSE)
